# 6,6′-Dimethyl-2,2′-[oxalylbis(aza­nedi­yl)]dipyridinium dichloride acetonitrile solvate

**DOI:** 10.1107/S1600536810033519

**Published:** 2010-08-25

**Authors:** Hui-Ling Hu, Pei-Chi Cheng, Chia-Jun Wu, Jhy-Der Chen

**Affiliations:** a Department of Chemical and Materials Engineering, Nanya Institute of Technology, Chung-Li, Taiwan; b Department of Chemistry, Chung-Yuan Christian University, Chung-Li, Taiwan

## Abstract

In the crystal structure of the title compound, C_14_H_16_N_4_O_2_^2+^·2Cl^−^·CH_3_CN, weak inter­molecular N—H⋯Cl hydrogen bonds are found between the H atoms bound to the pyridine and amine N atoms and the chloride anions. The asymmetric unit consits of one half cationic mol­ecule which is located on a centre of inversion, one chloride anion in a general position and one half acetonitrile mol­ecule which is located on a twofold axis. Because of symmetry, the C—H hydrogens of the acetonitrile solvent mol­ecule are disordered over two orientations.

## Related literature

For Ag(I) complexes incorporating *N*,*N′*-bis­(2-pyrid­yl)oxamide ligands which show one- and two-dimensional structures, see: Hsu & Chen (2004 ▶); Hu *et al.* (2004 ▶). For the synthesis of the starting reactant, see: Cheng *et al.* (2009 ▶).
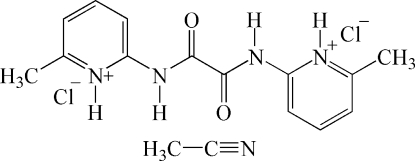


## Experimental

### 

#### Crystal data

C_14_H_16_N_4_O_2_^2+^·2Cl^−^·C_2_H_3_N
*M* *_r_* = 384.26
Monoclinic, 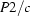

*a* = 10.6740 (19) Å
*b* = 8.7637 (5) Å
*c* = 10.370 (3) Å
β = 109.83 (2)°
*V* = 912.5 (3) Å^3^
*Z* = 2
Mo *K*α radiation
μ = 0.38 mm^−1^
*T* = 295 K
0.6 × 0.2 × 0.1 mm

#### Data collection

Bruker P4 diffractometer
Absorption correction: ψ scan (*XSCANS*; Siemens, 1995 ▶) *T*_min_ = 0.823, *T*_max_ = 0.922
2165 measured reflections
1619 independent reflections
1308 reflections with *I* > 2σ(*I*)
*R*_int_ = 0.023
3 standard reflections every 97 reflections  intensity decay: none

#### Refinement

*R*[*F* ^2^ > 2σ(*F* ^2^)] = 0.035
*wR*(*F* ^2^) = 0.094
*S* = 1.07
1619 reflections
118 parameters
H-atom parameters constrained
Δρ_max_ = 0.20 e Å^−3^
Δρ_min_ = −0.17 e Å^−3^

### 

Data collection: *XSCANS* (Siemens, 1995 ▶); cell refinement: *XSCANS*; data reduction: *XSCANS*; program(s) used to solve structure: *SHELXS97* (Sheldrick, 2008 ▶); program(s) used to refine structure: *SHELXL97* (Sheldrick, 2008 ▶); molecular graphics: *SHELXTL* (Sheldrick, 2008 ▶); software used to prepare material for publication: *SHELXTL*.

## Supplementary Material

Crystal structure: contains datablocks I, global. DOI: 10.1107/S1600536810033519/nc2195sup1.cif

Structure factors: contains datablocks I. DOI: 10.1107/S1600536810033519/nc2195Isup2.hkl

Additional supplementary materials:  crystallographic information; 3D view; checkCIF report

## Figures and Tables

**Table 1:** Hydrogen-bond geometry (Å, °)

*D*—H⋯*A*	*D*—H	H⋯*A*	*D*⋯*A*	*D*—H⋯*A*
N1—H1*A*⋯Cl^i^	0.86	2.14	2.9772 (16)	165
N2—H2*A*⋯Cl^i^	0.86	2.43	3.2057 (17)	150

Symmetry code: (i) 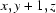.

**Fig. 1.**
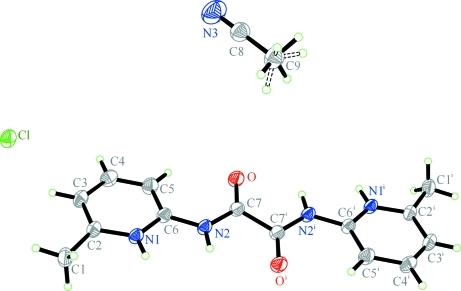
Crystal structure of the title compound with labeling and displacement ellipsoids drawn at the 30% probability level. Symmetry code: i = -x + 1,-y + 2,-z + 1. Disordering is shown as full and open bonds.

## supplementary crystallographic information

### Comment

Several Ag(I) complexes containg *N*,*N'*-bis(2-pyridyl)oxamide ligands have been prepared, which show one-dimensional and two-dimensional structures (Hsu, *et al.*, 2004; Hu, *et al.*, 2004). To investigate the steric effect of the alkyl groups on the structural type of such complexes, we have synthesized *N*,*N'*-bis(6-methyl-2-pyridyl)oxamide (Cheng, *et al.*, 2009) and reacted with metal salts. Within this project the crystal structure of the title compound was determined.

In the crystal structure the cationic molecules are almost planar and the O atoms are trans-oriented (Fig. 1). The *N*,*N'*-Bis(6-methyl-2-pyridinium)oxamide cations and the chloride anions are connected by weak intermolecular N—H···Cl hydrogen bonding (Tab. 1).

### Experimental

*N*,*N'*-bis(6-methyl-2-pyridyl)oxamide (0.30 g, 1.1 mmol) (Cheng, *et al.*, 2009) and CuCl_2_ (0.15 g, 1.1 mmol) were placed in a flask containing 10 ml CH_2_Cl_2_, which was refluxed for 8 h. The precipitate was then filtered and dried in vacuum. Coloress plate crystals of the title compound suitable for X-ray crystallography were obtained by slow evaporization of the solvent from a solution of the precipitate in CH_3_CN.

### Refinement

All the hydrogen atoms were placed into idealized positions (methyl H atoms allowed to rotate but not to tip) and constrained by the riding atom approximation with *C*—H = 0.93 — 0.96 Å, N—H = 0.86 Å and *U*_iso_(H) = 1.2 *U*_eq_(C, N) (1.5 for methyl H atoms).

### Crystal data

**Table:**

C_14_H_16_N_4_O_2_^2+^·2Cl^−^·C_2_H_3_N	*F*(000) = 400
*M**_r_* = 384.26	*D*_x_ = 1.399 Mg m^−^^3^
Monoclinic, *P*2/*c*	Mo *K*α radiation, λ = 0.71073 Å
Hall symbol: -P 2yc	Cell parameters from 28 reflections
*a* = 10.6740 (19) Å	θ = 4.7–12.5°
*b* = 8.7637 (5) Å	µ = 0.38 mm^−^^1^
*c* = 10.370 (3) Å	*T* = 295 K
β = 109.83 (2)°	Plate, colourless
*V* = 912.5 (3) Å^3^	0.6 × 0.2 × 0.1 mm
*Z* = 2	

### Data collection

**Table:**

Bruker P4 diffractometer	1308 reflections with *I* > 2σ(*I*)
Radiation source: fine-focus sealed tube	*R*_int_ = 0.023
graphite	θ_max_ = 25.0°, θ_min_ = 2.0°
ω scans	*h* = −12→12
Absorption correction: ψ scan (*XSCANS*; Siemens, 1995)	*k* = −10→1
*T*_min_ = 0.823, *T*_max_ = 0.922	*l* = −12→1
2165 measured reflections	3 standard reflections every 97 reflections
1619 independent reflections	intensity decay: none

### Refinement

**Table:**

Refinement on *F*^2^	Secondary atom site location: difference Fourier map
Least-squares matrix: full	Hydrogen site location: inferred from neighbouring sites
*R*[*F*^2^ > 2σ(*F*^2^)] = 0.035	H-atom parameters constrained
*wR*(*F*^2^) = 0.094	*w* = 1/[σ^2^(*F*_o_^2^) + (0.0401*P*)^2^ + 0.2279*P*] where *P* = (*F*_o_^2^ + 2*F*_c_^2^)/3
*S* = 1.07	(Δ/σ)_max_ < 0.001
1619 reflections	Δρ_max_ = 0.20 e Å^−^^3^
118 parameters	Δρ_min_ = −0.17 e Å^−^^3^
0 restraints	Extinction correction: *SHELXL97* (Sheldrick, 2008), Fc^*^=kFc[1+0.001xFc^2^λ^3^/sin(2θ)]^-1/4^
Primary atom site location: structure-invariant direct methods	Extinction coefficient: 0.0090 (15)

### Special details

**Table:**

Experimental. Refinement of *F*^2^ against ALL reflections. The weighted *R*-factor *wR* and goodness of fit *S* are based on *F*^2^, conventional *R*-factors *R* are based on *F*, with *F* set to zero for negative *F*^2^. The threshold expression of *F*^2^ > σ(*F*^2^) is used only for calculating *R*-factors(gt) *etc*. and is not relevant to the choice of reflections for refinement. *R*-factors based on *F*^2^ are statistically about twice as large as those based on *F*, and *R*- factors based on ALL data will be even larger.
Geometry. All esds (except the esd in the dihedral angle between two l.s. planes) are estimated using the full covariance matrix. The cell esds are taken into account individually in the estimation of esds in distances, angles and torsion angles; correlations between esds in cell parameters are only used when they are defined by crystal symmetry. An approximate (isotropic) treatment of cell esds is used for estimating esds involving l.s. planes.
Refinement. Refinement of F^2^ against ALL reflections. The weighted R-factor wR and goodness of fit S are based on F^2^, conventional R-factors R are based on F, with F set to zero for negative F^2^. The threshold expression of F^2^ > 2sigma(F^2^) is used only for calculating R-factors(gt) etc. and is not relevant to the choice of reflections for refinement. R-factors based on F^2^ are statistically about twice as large as those based on F, and R- factors based on ALL data will be even larger.

### Fractional atomic coordinates and isotropic or equivalent isotropic displacement parameters (Å^2^)

**Table:**

	*x*	*y*	*z*	*U*_iso_*/*U*_eq_	Occ. (<1)
Cl	0.23722 (6)	0.11398 (6)	0.09664 (5)	0.0569 (2)	
O	0.55710 (15)	0.81506 (16)	0.54656 (14)	0.0542 (4)	
N1	0.27012 (15)	0.78055 (18)	0.15297 (15)	0.0400 (4)	
H1A	0.2513	0.8715	0.1212	0.048*	
N2	0.39872 (16)	0.90108 (18)	0.34987 (15)	0.0424 (4)	
H2A	0.3653	0.9837	0.3073	0.051*	
N3	1.0000	0.6381 (4)	0.2500	0.0953 (12)	
C1	0.1214 (2)	0.7019 (3)	−0.0703 (2)	0.0596 (6)	
H1B	0.0933	0.6103	−0.1230	0.089*	
H1C	0.1660	0.7672	−0.1153	0.089*	
H1D	0.0450	0.7538	−0.0626	0.089*	
C2	0.2141 (2)	0.6624 (2)	0.0687 (2)	0.0443 (5)	
C3	0.2463 (2)	0.5175 (2)	0.1183 (2)	0.0521 (5)	
H3A	0.2118	0.4336	0.0627	0.062*	
C4	0.3305 (2)	0.4971 (3)	0.2514 (2)	0.0567 (6)	
H4A	0.3514	0.3985	0.2851	0.068*	
C5	0.3847 (2)	0.6201 (2)	0.3363 (2)	0.0522 (5)	
H5A	0.4406	0.6054	0.4262	0.063*	
C6	0.35325 (19)	0.7650 (2)	0.28310 (18)	0.0395 (4)	
C7	0.4915 (2)	0.9157 (2)	0.47672 (19)	0.0420 (5)	
C8	1.0000	0.7663 (4)	0.2500	0.0583 (8)	
C9	1.0000	0.9293 (4)	0.2500	0.0666 (9)	
H9B	1.0894	0.9658	0.2704	0.100*	0.50
H9A	0.9650	0.9658	0.3182	0.100*	0.50
H9C	0.9456	0.9658	0.1614	0.100*	0.50

### Atomic displacement parameters (Å^2^)

**Table:**

	*U*^11^	*U*^22^	*U*^33^	*U*^12^	*U*^13^	*U*^23^
Cl	0.0769 (4)	0.0307 (3)	0.0530 (3)	0.0030 (2)	0.0089 (3)	0.0052 (2)
O	0.0635 (9)	0.0404 (9)	0.0450 (7)	0.0124 (7)	0.0005 (7)	0.0022 (6)
N1	0.0498 (9)	0.0268 (8)	0.0401 (8)	0.0023 (7)	0.0112 (7)	0.0022 (6)
N2	0.0546 (10)	0.0297 (9)	0.0373 (8)	0.0057 (7)	0.0082 (7)	0.0012 (6)
N3	0.107 (3)	0.052 (2)	0.136 (3)	0.000	0.054 (3)	0.000
C1	0.0696 (15)	0.0438 (13)	0.0510 (12)	−0.0032 (11)	0.0017 (11)	−0.0076 (10)
C2	0.0499 (11)	0.0326 (10)	0.0494 (11)	−0.0034 (9)	0.0155 (9)	−0.0053 (8)
C3	0.0604 (14)	0.0300 (11)	0.0625 (13)	−0.0045 (9)	0.0166 (11)	−0.0053 (10)
C4	0.0689 (14)	0.0289 (10)	0.0680 (14)	0.0042 (10)	0.0178 (12)	0.0110 (10)
C5	0.0639 (13)	0.0361 (11)	0.0491 (11)	0.0044 (10)	0.0095 (10)	0.0071 (9)
C6	0.0465 (10)	0.0334 (10)	0.0382 (9)	0.0016 (8)	0.0140 (8)	0.0010 (8)
C7	0.0472 (11)	0.0398 (11)	0.0364 (10)	0.0059 (9)	0.0109 (8)	−0.0008 (8)
C8	0.0587 (19)	0.053 (2)	0.066 (2)	0.000	0.0248 (16)	0.000
C9	0.080 (2)	0.0492 (19)	0.068 (2)	0.000	0.0212 (18)	0.000

### Geometric parameters (Å, °)

**Table:**

O—C7	1.204 (2)	C2—C3	1.370 (3)
N1—C6	1.347 (2)	C3—C4	1.380 (3)
N1—C2	1.356 (2)	C3—H3A	0.9300
N1—H1A	0.8600	C4—C5	1.388 (3)
N2—C7	1.358 (2)	C4—H4A	0.9300
N2—C6	1.382 (2)	C5—C6	1.379 (3)
N2—H2A	0.8600	C5—H5A	0.9300
N3—C8	1.123 (5)	C7—C7^i^	1.545 (4)
C1—C2	1.486 (3)	C8—C9	1.429 (5)
C1—H1B	0.9600	C9—H9B	0.9600
C1—H1C	0.9600	C9—H9A	0.9600
C1—H1D	0.9600	C9—H9C	0.9600
			
C6—N1—C2	124.41 (17)	C3—C4—H4A	119.2
C6—N1—H1A	117.8	C5—C4—H4A	119.2
C2—N1—H1A	117.8	C6—C5—C4	117.98 (19)
C7—N2—C6	125.72 (16)	C6—C5—H5A	121.0
C7—N2—H2A	117.1	C4—C5—H5A	121.0
C6—N2—H2A	117.1	N1—C6—C5	118.82 (18)
C2—C1—H1B	109.5	N1—C6—N2	114.49 (16)
C2—C1—H1C	109.5	C5—C6—N2	126.69 (17)
H1B—C1—H1C	109.5	O—C7—N2	126.70 (18)
C2—C1—H1D	109.5	O—C7—C7^i^	122.0 (2)
H1B—C1—H1D	109.5	N2—C7—C7^i^	111.3 (2)
H1C—C1—H1D	109.5	N3—C8—C9	180.000 (2)
N1—C2—C3	117.77 (18)	C8—C9—H9B	109.5
N1—C2—C1	116.77 (18)	C8—C9—H9A	109.5
C3—C2—C1	125.46 (19)	H9B—C9—H9A	109.5
C2—C3—C4	119.4 (2)	C8—C9—H9C	109.5
C2—C3—H3A	120.3	H9B—C9—H9C	109.5
C4—C3—H3A	120.3	H9A—C9—H9C	109.5
C3—C4—C5	121.6 (2)		

Symmetry codes: (i) −*x*+1, −*y*+2, −*z*+1.

### Hydrogen-bond geometry (Å, °)

**Table:**

*D*—H···*A*	*D*—H	H···*A*	*D*···*A*	*D*—H···*A*
N1—H1A···Cl^ii^	0.86	2.14	2.9772 (16)	165.
N2—H2A···Cl^ii^	0.86	2.43	3.2057 (17)	150.

Symmetry codes: (ii) *x*, *y*+1, *z*.
